# Stable Atrial Sensing on Long-Term Follow Up of VDD Pacemakers

**Published:** 2006-10-01

**Authors:** Ashok Shah, Jairam Aithal, Dhiraj Narula, Prafulla Kerkar

**Affiliations:** 1 Ex-Registrar; 2 Ex-Senior Registrar; 3 Ex-Research Officer; 4 Professor and Head; Department of Cardiology, KEM Hospital, Mumbai, India

**Keywords:** VDD Lead, P wave Amplitude, Atrial Sensitivity, Atrioventricular synchronous Pacing

## Abstract

**Background:**

The hemodynamic advantages of maintaining AV synchrony through AV synchronous pacing are widely known as compared to single chamber pacing. DDD pacemaker implantation entails higher cost and is technically more challenging than the VDD pacemaker.

**Methods:**

Seventy one patients underwent VDD lead (Biotronik GmbH, St. Jude Medical and Medtronic Inc.) implantation at KEM hospital, Mumbai during a period of 3 years through subclavian, axillary and cephalic routes for degenerative, post-surgical or congenital high grade atrioventricular or complete heart block. They were followed up regularly for ventricular threshold and P wave amplitude of the floating atrial dipole.

**Results:**

Follow up data of almost 95% of patients is available for a period of 15.8 ± 6.7 months. P wave amplitude at implant was 2.1 ± 0.7mV and at follow up 1.1 ± 0.6mV with mean ventricular threshold of <0.5V at implant and <1V at follow-up.

**Conclusion:**

Implantation of a single lead VDD pacemaker is possible in all patients with symptomatic AV block and intact sinus node function without any technical complications. P wave sensing is reliable and consistent with floating atrial lead at an average follow up of 15.8 months, providing an excellent alternative to DDD pacemaker implantation.

## Introduction

The modalities of synchronous atrioventricular (AV) pacing in patients with symptomatic AV Block include VDD mode of pacing, besides the DDD mode which is accepted as a gold standard modality. Technical difficulties in placing atrial lead, higher cost of the procedure due to need for second (atrial) lead are the limitations of DDD mode of pacing. Here we propose to evaluate the stability of single lead VDD pacemaker in terms of atrial signal detection via floating atrial electrode.

## Methods

### Patients

Seventy one patients with high degree AV block underwent implantation of a VDD pacemaker from December 2002 through November 2005 at KEM Hospital, Mumbai. The patients who underwent VDD pacemaker implantation were evaluated by 24 hours ECG monitoring / chronotropic sinus node response to stress for sinus node function. Patients with sinus node dysfunction and / or atrial arrhythmias were not eligible for VDD pacemaker implantation.

The indications for VDD pacing  were degenerative  high grade AV block in 66, complete heart block (CHB) following aortic valve replacement in 3, intracardiac repair for partial AV canal defect in 1 and congenital CHB in 1.

### Pacemaker system

The Solox quadripolar single VDD Pacing Lead electrode (Biotronik GmbH,Germany), model 1368 VDD Pacing Lead (St. Jude Medical,USA) and  model 5038 CapSure®VDD Pacing Lead (Medtronic Inc.,USA) were implanted in 48,13 and 10 patients respectively. Leads with separation distance of 13cm and 11cm between ventricular tip and atrial dipole were used in 68 and 3 patients respectively.

### Implantation technique

The implantation of single VDD lead (6.3 Fr) was performed by subclavian vein puncture in 52 patients, by axillary vein puncture in 11 patients and by cephalic vein cut down in 8 patients. Axillary vein puncture was guided by ipsilateral contrast venogram. After the placement of the electrode tip into the right ventricle, the ventricular pacing threshold, the amplitude of R wave (wherever feasible), the lead impedance and the slew rate were determined. P wave amplitude was measured during normal and deep breaths and during cough after the atrial dipole was positioned in visually optimal position. By adjusting the intravascular length of the lead, the best atrial sensing was determined. The lead position and parameters were rechecked after coughing and diaphragmatic pacing was ruled out before fixing the leads. Eventually the lead was attached to the pulse generator and the pulse generator was implanted in the prepectoral pocket. Upper and lower tracking rate limits were determined and atrial sensitivity and paced AV delay were programmed to maximize AV synchronous pacing.

### Follow up

Patients were discharged 2 days post implantation after confirming the lead position on penetrated chest roentgenograms and confirming the lead parameters including P wave amplitude. If required, the reprogramming was done to adjust atrial sensitivity and to optimize AV synchronous pacing. They were reexamined for the status of the wound at the site of pacemaker implantation after 10 ± 2 days. The pacemaker was interrogated after 2 to 3 months of follow up and subsequently at every 6 monthly follow-up visits.

### Statistics

Values obtained were analyzed using SPSS software for Windows to obtain mean and standard deviation (SD). The expressed values in the text / table are mean ± 1 SD.

## Results (Table 1 and 2)

Out of 71 patients who underwent VDD pacemaker implantation 1 patient expired of acute coronary event and 3 others haven't followed up subsequently hitherto. The duration of follow up for the rest 67 subjects has been 15.8 ± 6.7 months. Two patients experienced loss of atrial sensing on day 1 and at 9 months follow up respectively. The pacing mode was switched over from VDD to VVI in both. Two months later, the first patient had good P wave amplitude and the mode was switched over to VDD again. Another 6 months later, the P wave height was 1.9 mV (3 mV at implant) with the ventricular threshold of 0.9 V (0.5 V at implant), thereby obviating repositioning of the lead.

Another patient who underwent VDD pacemaker implantation for CHB expired 6 days after implantation of refractory heart failure following an acute coronary event sustained on day 2 post procedure. The pacing parameters throughout the turbulent postoperative period were satisfactory with P wave amplitude of 3 mV and ventricular threshold of 0.4 V.

## Discusssion

The hemodynamic advantages of maintaining AV synchrony through AV synchronous pacing are widely known as compared to single chamber pacing. DDD pacemaker implantation is technically more challenging in terms of the need to insert a second lead into the atrium for sensing and pacing the atria. This also increases the cost of the procedure and the total fluoroscopy time of the procedure. A further disadvantage is high complication rate with atrial (2.9 % in Parromet and Bernstein series [1], 11 % Brownlee series [2]). The complications include lead dislocation, loss of atrial sensing, lead fracture, etc.  Diminution of atrial sensing threshold is seen with walking, running and exercise in standing posture (Froehlig et al series [3,4],  Zitzmann et al series [5]).

Single lead VDD pacing offers an opportunity for AV synchronous pacing with the simplicity of VVI implantation procedure and without complexity of atrial electrode.

Out of 67 cases that followed up with us, just one case has been found to have loss of atrial sensing at follow up visit. In another case there was loss of atrial sensing on day 2 that reverted spontaneously at subsequent follow up visit without entailing repositioning of the lead. Thus, only one patient was found to have loss of atrial sensing at 9 months of follow up. The ultimate goal of VDD pacing is a chronically stable P wave sensing. Studies have shown atrial sensing performance of > 94 % in the majority of them so far [6-9]. Though it is not predictable the loss of AV synchrony can be minimized by achieving a large and a stable intraoperative P wave amplitude, by positioning the atrial dipole in high right atrium and keeping the programmable atrial sensitivity to as low a value as possible with the available VDD lead (0.1mV in our study) [10].

None of our patients has developed atrial arrhythmias on follow up. In one of the multicenter study 2.1 % of the patients treated with VDD developed atrial fibrillation (AF) [11].  The incidences in two other studies are 7 % and 6.4 % respectively [8,12]. This is accounted by a large number of patients with organic heart disease and patients with Aortic / Mitral valve replacement who needed VDD pacemaker in these   studies. Four patients with organic heart disease have undergone VDD pacemaker implantation but haven't had atrial fibrillation so far.

Although rare, sick sinus syndrome may develop in patients with AV block (binodal disease). As the atrium can't be paced, this may be the major drawback of VDD mode [8].

However studies are tried to stimulate atria using overlapping biphasic impulse (OLBI) stimulation waveform delivered via an atrial floating electrode but this is limited by high pacing threshold due to unstable atrial floating lead position and also by phrenic nerve stimulation which is often observed with high right atrial position of the VDD lead [13].

As of now, a careful patient selection as like excluding patients with sinus node dysfunction and intermittent / persistent atrial fibrillation/flutter is required.

## Study Limitations

All the patients who were considered for VDD permanent pacemaker in this study were evaluated for the sinus node function either by 24 hour Holter or Exercise ECG before implantation.  They have not been evaluated post implantation through Holter monitoring which helps to assess the variation in the P wave amplitude during routine work, postural changes and moments of unusual physical and non-physical stress. It is in these situations that the best of the utilities of VDD pacing is assessed viz.  Preservation of AV synchrony and thereby retention of the most physiological rate response to stress even with a single pacing lead system.

Because the working amplitude of P wave varies with posture and exercise it can be reproducibly assessed in a controlled manner through Tread Mill Test (TMT). The episodic occurrence of VVI pacing during the test suggests loss of atrial sensing during that particular phase of the test. In this study, we have not resorted to TMT on follow up which is advisable for a comprehensive assessment of the stability of atrial dipole through actual period of day to day work.

## Conclusion

It has been shown in the present study that the implantation of a new VDD single lead pacemaker was possible in all patients with symptomatic AV block and intact sinus node function without any technical complication of the pacemaker system. P wave sensing was reliable and consistent with floating atrial lead at an average follow up of 15.8 months as assessed from the retrieval of stored histograms. Post implantation, 24 hour Holter and TMT give comprehensive assessment of the stability of the floating atrial electrodes. The VDD pacing offers an excellent alternative to conventional DDD stimulation.

## Figures and Tables

**Table 1:**
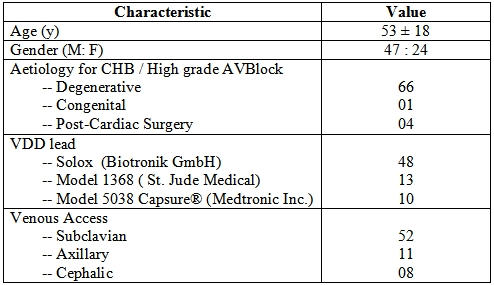
Study Profile

**Table 2:**
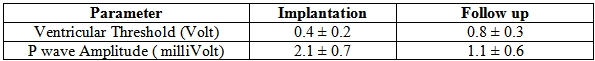
VDD Lead Parameters
